# Electrophysiological correlates of masked face priming

**DOI:** 10.1016/j.neuroimage.2007.12.003

**Published:** 2008-04-01

**Authors:** R.N. Henson, E. Mouchlianitis, W.J. Matthews, S. Kouider

**Affiliations:** a MRC Cognition and Brain Sciences Unit, 15 Chaucer Road, Cambridge CB2 7EF, England, UK; b Department of Psychology, Warwick University, UK; c Laboratoire des Sciences Cognitives et Psycholinguistique, CNRS/EHESS/DEC-ENS, Paris, France; d Cognitive Neuroimaging Unit, INSERM/SHFJ/CEA, Orsay, France

## Abstract

Using a sandwich-masked priming paradigm with faces, we report two ERP effects that appear to reflect different levels of subliminal face processing. These two ERP repetition effects dissociate in their onset, scalp topography, and sensitivity to face familiarity. The “early” effect occurred between 100 and 150 ms, was maximally negative-going over lateral temporoparietal channels, and was found for both familiar and unfamiliar faces. The “late” effect occurred between 300 and 500 ms, was maximally positive-going over centroparietal channels, and was found only for familiar faces. The early effect resembled our previous fMRI data from the same paradigm; the late effect resembled the behavioural priming found, in the form of faster reaction times to make fame judgments about primed relative to unprimed familiar faces. None of the ERP or behavioural effects appeared explicable by a measure of participants’ ability to see the primes. The ERP and behavioural effects showed some sensitivity to whether the same or a different photograph of a face was repeated, but could remain reliable across different photographs, and did not appear attributable to a low-level measure of pixelwise overlap between prime and probe photograph. The functional significance of these ERP effects is discussed in relation to unconscious perception and face processing.

A stimulus can be made difficult if not impossible to perceive by presenting it briefly, preceded and succeeded by “forward” and “backward” pattern masks: so-called “sandwich masking”. Numerous studies have shown that such “invisible” stimuli can nonetheless affect behavioural responses to subsequent probe stimuli presented shortly after the backward mask: so-called “subliminal priming”. For example, if the invisible stimulus (the “prime”) matches the probe on some perceptual dimension, decisions about the probe can be facilitated in either accuracy or reaction time. This priming effect has been used for several decades in behavioural research to make inferences about the nature and extent to which stimuli can be processed in the absence of awareness (see Kouider and Dehaene, 2007, for a review).

In a recent fMRI experiment using sandwich-masked faces (Kouider et al., in press), we demonstrated reduced BOLD responses for primed vs. unprimed stimuli – so-called “repetition suppression” (Grill-Spector et al., 2006) – in regions of the ventral visual-object processing stream. More specifically, occipital and fusiform “face areas” (OFA and FFA, respectively), which were generally responsive to faces, showed a smaller BOLD response when the prime and probe were images of the same person’s face (even when the image differed in size, and when the image was a different photograph of the same face), relative to the unprimed situation where the prime and probe were images of two different persons. This occurred in conjunction with faster reaction times to make a fame judgment about photographs of famous faces. Importantly, both the fMRI and the behavioural priming effects remained when taking into account a measure of prime visibility for each participant. Consistent with previous imaging work using words (Dehaene et al., 2001, 2004) and objects (Eddy et al., 2007), our study therefore suggested that subliminal faces can be processed to a degree of perceptual abstraction, and that neural activity in OFA and FFA does not always correlate with visual awareness.

In the present study, we extend this previous work by investigating the temporal characteristics of subliminal face priming, through an EEG version of our fMRI experiment. In particular, we wished to address two main issues. First, one puzzle regarding our fMRI data is that repetition suppression appeared equivalent for both familiar (famous) and unfamiliar (nonfamous) faces, whereas behavioural priming was much greater for familiar than unfamiliar faces (indeed, only marginal for unfamiliar faces). This suggests that there are contributions to behavioural performance in this task that are additional to any contributions arising from activity in ventral temporal cortex. Second, the BOLD response, which represented the summed activity to prime, probe, and mask, was unable to discern the temporal evolution of the priming effect(s), allowing the possibility that the BOLD repetition suppression reflected neural activity occurring after the (facilitated) behavioural response. EEG data offer the opportunity to test whether neural activity associated with subliminal priming arises relatively “early”, perhaps reflecting mainly feedforward, stimulus-driven activity, or relatively “late”, perhaps reflecting recurrent activity between brain regions (Henson, 2003).

The basic design is shown in Fig. 1. A forward mask consisting of a number of superimposed, inverted faces was followed by the prime face for 50 ms, a backward mask for another 50 ms, and the probe face for 700 ms. Note that the prime was 80% smaller than the probe to avoid exact pixelwise overlap. Participants made a speeded fame judgment to the probe. They were not informed of the presence of the prime until a subsequent debriefing stage, in which their ability to see the prime was assayed. There were 6 different trial types in the main priming experiment, conforming to a 2 × 3 factorial design with factors: familiarity (Famous vs. Nonfamous), and prime condition (Same-View, where prime and probe were identical photographs; Different-View, where prime and probe were different photographs of the same person; and Unprimed, where photographs were of different persons, though always of the same response category, i.e., both famous or both nonfamous). Behavioural priming was indexed by reaction times (RTs) for Same-View and Different-View conditions relative to the Unprimed condition.

## Materials and methods

### Participants

Fourteen volunteers gave written consent to participate in the study, reporting themselves to be in good health, with no history of neurological illness. The data from two volunteers were not analysed because of EEG artefact or an inability to recognise enough of the famous faces, leaving 3 men and 9 women, aged 19–29, three left-handed. The study was approved by Cambridge University Psychological Ethics Committee (reference CPREC 2005.08).

### Materials

The stimuli were greyscale photographs of faces. A subset of 40 famous and 40 nonfamous faces were selected from a set used previously (Eger et al., 2005) as those most often recognised and not recognised respectively by young British adults. Half were male; half were female. All photos were matched for image size, and cropped to show face and hair only.

There were two different photos of each face. There was no explicit control of the differences across the two views of each face: The two photographs could be taken from different perspectives (though the majority were between frontal and three-quarter views), involve different facial expressions and/or differences in lighting conditions, or hairstyles. There was little difference across the two photographs in the age of the person. Unfamiliar faces were matched to famous counterparts with respect to gender and approximate age. An attempt was also made to homogenise the stimuli with respect to average luminance and contrast. The mean luminance, as defined by mean pixel intensity 0–1, averaging across view, was 0.33 (SD = 0.054) and 0.32 (SD = 0.056) for famous and nonfamous faces, respectively, a difference that was not significant, *t*(78) = 0.63, *p* = .53 (two-tailed). The mean contrast, as defined by the SD of the intensity across pixels, was 0.30 (SD = 0.027) and 0.31 (SD = 0.035), respectively, a difference that was also not significant, *t*(78) = 1.27, *p* = .21 (two-tailed).

**Fig. 1:**
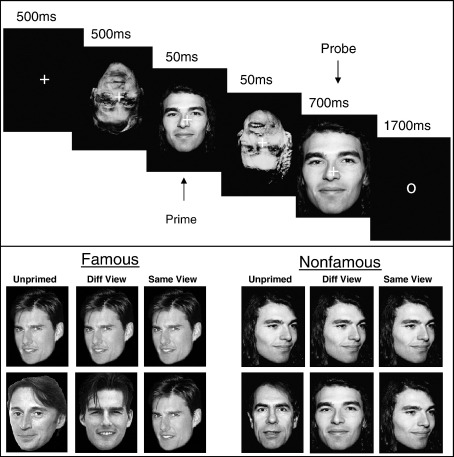
Trial procedure and experimental design.

Eighty masks were created by overlaying 4 inverted faces (half famous, half female). In order to minimise pixel overlap with the probe face, the prime was scaled to be 80% smaller than the probe (masks received the same size reduction for masking improvement reasons). See Fig. 1 for examples.

The 480 trials (80 per condition) were split into two sessions by a short rest break. Each face was presented once every third of a session (i.e., within a permuted set of 80 trials) and, across a whole session, appeared in each of the three conditions. The specific photograph used for a given probe face was swapped across sessions. The assignment of face images to priming conditions was thus counterbalanced within participants, i.e., the set of prime and probe faces was matched across each level of the priming factor (though of course different across the familiarity factor), which is important in short SOA designs to ensure that any priming-related modulations of the ERP data following probe onset are not consequences of differences in the prior prime stimulus.

### Procedure

Stimuli were presented on a CRT screen against a black background and with a white fixation cross in the centre of the photograph (Fig. 1). The start of a trial was indicated by the appearance of a fixation cross for 500 ms, followed by a forward mask of 500 ms, the prime face for 50 ms, a backward mask (different from the forward mask) for 50 ms, and the probe for 700 ms. The prime and backward mask durations were locked to the onset and offset of 3 screen refreshes (using a 60 Hz refresh rate) using the Cogent freeware (http://www.vislab.ucl.ac.uk/Cogent/) running in Matlab6.5 (The Mathworks, http://www.mathworks.com/products/matlab). At the offset of the probe face, the central cross was replaced with a central circle, which remained on the screen for 1700 ms until the next trial started. Each of the two main experimental sessions lasted approximately 12 min.

The participant sat approximately 140 cm from the screen, with the probe stimuli subtending a vertical and horizontal visual angle of approximately 3.2° and 4.1°, respectively (or 2.6 and 3.3 in the case of the smaller mask and prime stimuli). The participant’s task was to decide, as quickly as possible, whether or not each (probe) face belonged to a famous person. They indicated their response with either their left or right index finger, counterbalanced across participants. The prime and probe were always both famous or both nonfamous, so that any difference between the Same/Different-View conditions and the Unprimed condition could not be attributed to priming of a response (rather than of a specific stimulus). Participants were told that some strange pictures of scrambled faces would appear before the face, but to ignore these and concentrate on fixating on the central cross and responding to the final (probe) face. They were not informed about the presence of the prime faces. Each participant received 80 practice trials (approximately 4 min), which included one presentation of each face, and many examples of each condition.

In a final session, a prime “visibility test” was administered, consisting of 156 trials and lasting 8 min. Participants were now informed of the presence of prime faces, and asked to guess whether or not they were famous. The stimulus presentation conditions were identical to the main experiment, apart from the composition of the trials. For one half of trials, the fame of the prime and probe matched, as in the main experiment (and these were divided equally among the six conditions of the main experiment, i.e., famous/nonfamous crossed with same view, different view, and unprimed). For the other half of visibility trials, the fame of the prime and probe faces differed. This meant that the fame of the probe could not be used to predict the fame of the prime. Indeed, participants were told to ignore the fame of the probe face because it was not correlated with the fame of the prime face. Their judgment for each prime was conditionalised on an estimate of whether they actually knew that face was famous, derived from their fame judgments to that face when it appeared as a (clearly visible) probe in the main priming task (i.e., Sessions 1–2). Only the first 64 trials were analysed, to reduce learning effects across the visibility test and for consistency with Kouider et al. (in press). On average, these 64 trials comprised 30 nonmatch and 30 match trials (after removing invalid responses), with the match trials distributed equally over the conditions in the main experiment.

### EEG acquisition

The electroencephalogram (EEG) was measured in an electrically and acoustically shielded booth at the MRC Cognition and Brain Sciences Unit. Data were recorded from 63 Ag/AgCl electrodes mounted on an electrode cap (Easycap, Falk Minow Services, Herrsching-Breitbrunn, Germany, http://www.easycap.de/easycap) using SynAmps amplifiers (NeuroScan Labs, Sterling, USA), arranged according to the extended 10/20 system. Data were acquired with a sampling rate of 500 Hz. Recording reference for the EEG channels was Cz. The EOG was recorded bipolarly through electrodes placed above and below the left eye (vertical) and at the outer canthi (horizontal). Impedances were generally less than 6 kOhms. Recordings were amplified with a bandwidth of 0.1–100 Hz and digitised with 16 bits (0.168 μV/bit).

### Behavioural analysis

For the main priming sessions, trials with reaction times (RTs) less than 200 ms or greater than 1000 ms were rejected (as in Kouider et al., in press). An omnibus, repeated-measures Analyses of Variance (ANOVA) on mean RTs was followed by two more focussed 2 × 2 ANOVAs. The first “global priming” ANOVA crossed familiarity (familiar vs. unfamiliar) with repetition (unprimed vs. primed, collapsing across same vs. different view). The second 2 × 2 ANOVA on “view effects” crossed familiarity with view-change (same vs. different views). Significance was defined as a *p*-value below .05. Significant effects were only reported in the absence of significant higher-order interactions. Subsequent planned, pairwise tests were used to assess the reliability of priming in each of the four critical conditions (vs. the unprimed baseline). All such *t*-tests were two-tailed, but not corrected for the multiple planned comparisons.

### ERP analysis

Preprocessing was automated using EEG functions from SPM5 (http://www.fil.ion.ucl.ac.uk/spm); statistical analysis was performed using additional code written in Matlab (The Mathworks,http://www.mathworks.com/products/matlab) by the first author. The continuous EEG data for each session were epoched from − 100 ms to 700 ms, where 0 ms corresponds to the onset of the prime, baseline-corrected relative to the 100 ms pre-prime period, and combined across the two sessions. The data were subsequently resynchronised to the onset of the probe (i.e., − 200 to 600 ms). Only trials with correct responses were epoched. Epochs in which the signal from any channel (including EOG) exceeded 120 μV were removed (median number of trials = 8; range = 4–75), and confirmed by visual inspection. The data were rereferenced to the average over all EEG channels. The ERP for each condition was created by averaging over trials.

### Space × time SPM analysis

Given that we had few a priori predictions for when (within the epoch) or where (over channels) masked face repetition effects might arise, we first adopted a mass univariate approach in which *F*-tests were performed at every point in a 3D image of channel space × time. The 2D channel space was created by a spherical projection of the standardised Easycap channel locations onto a plane followed by a linear interpolation to a 32 × 32 pixel grid; the time dimension consisted of the 401 2 ms samples in the epoch. *F*-tests corresponding to the main effect of priming condition, main effect of familiarity and their interaction, were performed within a GLM using a pooled error over the 6 conditions. The nonsphericity across conditions (owing to repeated measures from the same subject) was estimated using Restricted Maximal Likelihood, and used to pre-whiten the model and data (Friston et al., 2002). *F*-values were subsequently converted to *Z*-values. The resulting statistical parametric map (SPM) was family-wise error (FWE) corrected for multiple comparisons using Random Field Theory (Worsley et al., 1995; Kiebel and Friston, 2004), with the estimated Gaussian FWHM smoothness being approximately 6 pixels in space and 5 samples in time.

### Time window analysis

Having identified time windows of interest from the SPM analyses, additional ANOVAs were performed on the mean amplitude (with respect to mean pre-stimulus baseline) during these time windows, with the factors of familiarity and priming being supplemented by further channel factors (see Results). As with the behavioural analyses, two such ANOVAs were performed: one for global repetition effects and one for view effects. For ANOVA effects involving more than 1 *df*, a Greenhouse–Geisser correction for nonsphericity was used.

## Results

### Behavioural results

Less than 3% of responses on average were rejected due to the response window (see Materials and methods) (range = 0–16%), and less than 6% of the remaining trials were incorrect fame judgments (range = 1–15%), i.e., accuracy was close to ceiling. Reaction times (RTs) for correct judgments are shown in Table 1. The following RT and ERP analyses are restricted to “familiar” and “unfamiliar” face trials (i.e., famous and nonfamous faces correctly classified for each individual participant).

The 3 × 2 omnibus ANOVA showed reliable main effects of priming condition, *F*(1.96,21.5) = 9.49, *p* < .001, and of familiarity, *F*(1,11) = 22.4, *p* < .001, and an interaction that approached significance, *F*(1.99,21.9) = 2.97, *p* = .07. The main effect of familiarity reflected faster RTs for familiar faces. The effect of priming condition was investigated by the two planned comparisons of (1) “global” priming (collapsing Same- and Different-View conditions) and (2) “view” effects (contrasting Same- and Different-View conditions; see Materials and methods). The 2 × 2 ANOVA on “global” priming showed a reliable familiarity-by-priming interaction, *F*(1,11) = 6.09, *p* < .05, with greater priming for familiar than unfamiliar faces. The 2 × 2 ANOVA on “view effects” showed a reliable main effect of view, *F*(1,11) = 5.99, *p* < .05, though no interaction with familiarity, reflecting greater priming for same than different views. Planned comparisons of the amount of priming for each condition (i.e., RT reductions relative to the unprimed condition) showed reliable priming for Same-View Familiar faces, *M* = 20.9 ms, T11 = 3.78, *p* < .005, and for Different-View Familiar faces, *M* = 12.6 ms, T11 = 2.57, *p* < .05; priming for Same-View Unfamiliar faces was marginal, *M* = 7.36 ms, T11 = 1.98, *p* = .07.

**Fig. 2:**
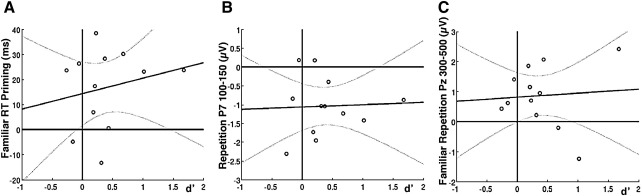
Regression of global repetition effects against *d*′ across participants, for (A) Behavioural RT priming for familiar faces, (B) ERP repetition effect for familiar and unfamiliar faces from 100 to 150 ms on channel P7, and (C) ERP repetition effect for familiar faces from 300 to 500 ms on channel Pz. Each participant is a point; dark line represents linear fit; dashed curves reflect 95% confidence interval of fit. Importantly, the *y*-intercept is significantly different from zero in each case, suggesting that *d*′ cannot (in simple terms) explain the behavioural or ERP effects (see text).

Debriefing participants before the prime visibility test revealed that a few noticed an occasional flash of an upright face prior to the main face, but did not think much of it, and did not notice any repetitions of the same image or person. The subsequent forced-choice fame-judgment task on the primes confirmed that our masking method rendered the primes difficult to see, as performance was close to chance (*M* = 56.3%, SD = 8.5%), with a *d*′ close to zero (*M* = 0.39, SD = 0.54).^1^ Although *d*′ was significantly above zero (T11 = 2.50, *p* < .05), priming was still reliable when the prime discrimination task was extrapolated to null performance (Fig. 2A; see Greenwald et al., 1995; Hannula et al., 2005). More specifically, regression of each participant’s priming for familiar faces (given that priming was less reliable for unfamiliar faces), averaging across view, against their *d*′ showed a reliable positive intercept, *M* = 14.3 ms (95%CI = [1.3–27.4] ms), as also the case in Kouider et al. (in press). In other words, even if primes could occasionally be seen when a participant was explicitly told of their presence and asked to categorise them, this would not seem able to explain the amount of behavioural priming they showed in the preceding sessions.

### ERP results

#### Preview

The ERPs at two channels are shown in Figs. 4 and 5, respectively, graphed separately for familiar (upper right; blue) and unfamiliar (lower right; red) faces. The conventional P1/N1 components associated with onset of the probe are difficult to see because the waveforms represent the combination of evoked responses to prime onset (− 100 ms), backward mask onset (− 50 ms), and probe onset (0 ms). Note that because every face appeared as prime and/or probe in every condition (for a given fame category), and given that masks were randomly assigned to trials for each participant, differences in the evoked response between priming conditions cannot be simply explained by differences in the nature of the visual stimuli preceding probe onset (rather, such differences reflect the *relationship* between prime and probe). It is also noteworthy that at the posterior sites (Fig. 4), there is a negative deflection around 170 ms after the *prime* onset, which resembles the N170 effect believed to index face processing (Bentin et al., 1996; Botzel et al., 1995). This is consistent with experiments by Jeffreys (1996), in which he found only a single vertex positive potential (VPP; most likely the vertex analogue of the N170) for two faces presented in quick succession (50 ms SOA), which resembled that seen when the first face was presented alone. In other words, the subjectively masked first face evokes a face-specific response, but the perceptually dominant second face does not.

### Space × time SPM results

**Fig. 3:**
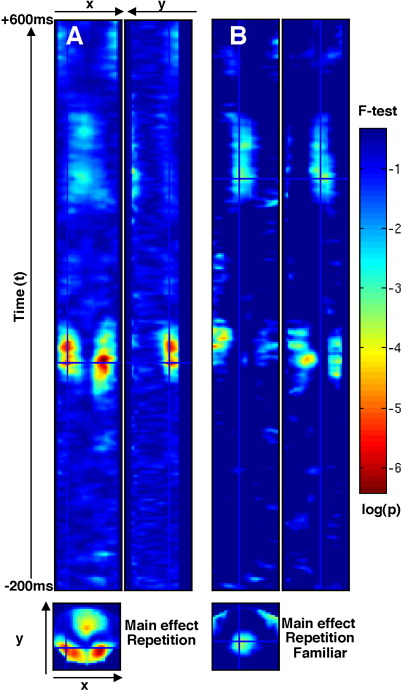
Two-tailed, unthresholded space-time Statistical Parametric Maps (SPMs) for (A) the main effect of priming, with crosshair located on left posterior maximum at 122 ms (see Table 2), and (B) the simple effect of priming for familiar faces, with crosshair located on centroparietal maximum at 378 ms. The three images in each panel represent orthogonal planes (*x*-*t*, *y*-*t*, and *x*-*y*) through the 3D image at the location of the crosshair.

The SPM for the main effect of priming condition showed a cluster between approximately 100 and 150 ms over bilateral temporoparietal pixels and midline occipital pixels (Fig. 3A) that survived correction for multiple comparisons (with various submaxima shown in Table 2). This “early” effect reflected more negative potentials for the primed conditions relative to the unprimed condition. The main effect of familiarity did not emerge until approximately 300 ms and included a large central cluster showing more positive potentials for familiar than unfamiliar faces (accompanied by more negative potentials in clusters of peripheral pixels).

No clusters survived correction for the familiarity-by-priming interaction. However, given the stronger behavioural priming effects for familiar than unfamiliar faces, the simple effect of priming was also performed for familiar faces alone. In addition to the above early repetition effect, this simple effect contrast revealed a centroparietal cluster maximal at 378 ms (Fig. 3B) that survived correction for the (orthogonal) main effect of familiarity. This “later” repetition effect reflected more positive potentials for primed familiar faces than unprimed familiar faces. Topographic movies of these “early” and “late” repetition effects can be observed here: http://www.mrc-cbu.cam.ac.uk/~rh01/early-rep.avi and http://www.mrc-cbu.cam.ac.uk/~rh01/late-rep.avi. More detailed analyses of the two effects follow below.

Time-frequency SPMs (using Morlet wavelets) were also calculated (analogous to Henson et al., 2005), to check for induced repetition effects, but no additional results were found.

### Time window results

Given the significant effects identified in space and time by the above SPM analyses, more focussed (and more conventional) tests were performed on the mean amplitude within two time windows – 100–150 ms (“early repetition effect”) and 300–500 ms (“late repetition effect”) – for the channels showing the maximal effects within these windows in the SPM analysis.^2^

#### 100–150 ms ERP

**Fig. 4:**
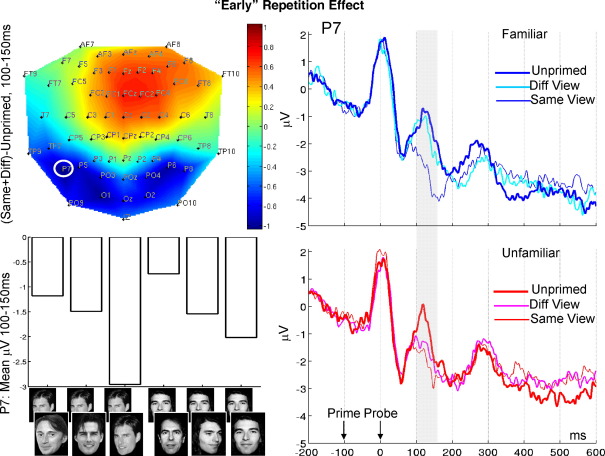
Early ERP repetition effect. Shown is the scalp topography of the global repetition effect from 100 to 150 ms (top left), the ERPs for familiar face conditions (top right) and for unfamiliar face conditions (bottom right) from Channel P7 (circled in topography), and the mean amplitude from 100 to 150 ms across conditions for this channel (bottom left).

The 2D topography of the global repetition effect, averaged across the early time window, is shown in Fig. 4 (top left). A more negative-going deflection was seen for primed faces at bilateral temporoparietal channels. Channels P7 and O1 were selected from the coordinates of the SPM analysis, as well as their right-hemisphere homologues P8 and O2. These channels were factorised as Left/Right by Parietal/Occipital in subsequent ANOVAs.

The omnibus ANOVA showed a reliable interaction between familiarity and priming condition, *F*(1.66,18.3) = 6.36, *p* < .05, but no reliable interactions with the two channel factors. This interaction was explored further with the planned comparisons of global repetition and of view. The ANOVA on the global repetition effect showed a highly reliable main effect of repetition, *F*(1,11) = 22.4, *p* < .001. This effect was found for both familiar, *F*(1,11) = 7.58, *p* < .05, and unfamiliar, *F*(1,11) = 26.0, *p* < .001, faces.

The ANOVA on view effects showed a reliable familiarity-by-view interaction, *F*(1,11) = 13.7, *p* < .005. Follow-on ANOVAs showed a reliable view effect for familiar, *F*(1,11) = 72.8, *p* < .001, but not unfamiliar, *F*(1,11) = 2.31, *p* = .16, faces. This reflected greater repetition effects for Same- than Different-View familiar faces.

Finally, planned comparisons of the repetition effect for each condition showed reliable main effects of repetition for Same-View Familiar faces, and both Same-View and Different-View Unfamiliar faces, *F*s > 19, *p* < .001. There was no reliable repetition effect for Different-View Familiar faces, though there was a reliable repetition-by-hemisphere interaction, *F*(1,11) = 5.63, *p* < .05. This appeared to reflect a more negative-going repetition effect on left than right channels, but was difficult to interpret, given that no repetition effects were reliable when analysing left and right hemisphere channels separately, *F*s < 1.1, *p*s > .33.^3^

In summary, the general pattern for the early ERP effect was a posterior temporal/parietal/occipital negative deflection for repetitions of same-view faces relative to two different faces (see e.g., at channel P7 in Fig. 4, bottom left), regardless of whether those faces were familiar or unfamiliar. For unfamiliar faces, a significant repetition effect was also seen across different views of a repeated face. Yet for familiar faces, there was no evidence for a repetition effect across different views. This pattern across conditions is different from that in the behavioural data (Table 1). For example, behavioural priming for Different-View faces was found only for Familiar faces, yet an ERP repetition effect for Different-View faces was found only for Unfamiliar faces. One possibility is that this early repetition effect reflects “low-level” visual overlap between prime and probe, which does not contribute appreciably to the time taken to judge a probe face as famous. This possibility is pursued later.

#### 300–500 ms ERP

**Fig. 5:**
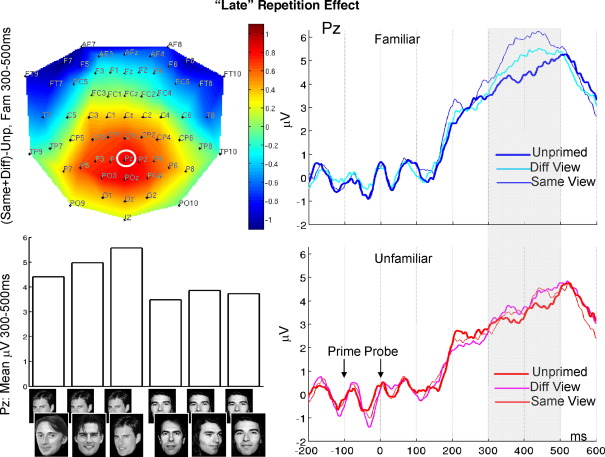
Late ERP repetition effect. Shown is the scalp topography of the global repetition effect for familiar faces from 300 to 500 ms (top left), the ERPs for familiar face conditions (top right) and for unfamiliar face conditions (bottom right) from Channel Pz (circled in topography), and the mean amplitude from 300 to 500 ms across conditions for this channel (bottom left).

The 2D topography of the global repetition effect for familiar faces, averaged across the late time window, is shown in Fig. 5 (top left). Primed familiar faces led to more positive deflections than unprimed familiar faces over centroparietal sites. Channels P1, Pz, and P2 were selected from the coordinates of the SPM analysis, and entered as a single factor in subsequent ANOVAs.

The omnibus ANOVA showed a reliable main effect of priming condition *F*(1.76,19.4) = 6.54, *p* < .01, plus a borderline familiarity-by-priming-by-channel interaction, *F*(3.3,36.3) = 2.66, *p* = .058. This interaction was explored further with the planned comparisons of global repetition and of view. The ANOVA on the global repetition effect showed a reliable main effect of repetition, *F*(1,11) = 13.58, *p* < .005, which subsequent ANOVAs showed was reliable for familiar, *F*(1,11) = 9.79, *p* < .01, but not for unfamiliar, *F*(1,11) = 2.42, *p* = 0.15, faces.

The ANOVA on view effects showed a familiarity-by-view interaction that approached significance, *F*(1.48,16.2) = 3.08, *p* = .08. Follow-on ANOVAs showed a reliable view effect for familiar, *F*(1,11) = 5.50, *p* < .05, but not unfamiliar, *F* < 1, faces. This reflected a greater repetition effect for Same- than Different-View Familiar faces.

The planned comparisons of the repetition effect for each condition showed a reliable repetition effect for Same-View Familiar faces *F*(1,11) = 11.8, *p* < .01, and a repetition effect for Different-View Familiar faces that approached significance, *F*(1,11) = 4.10, *p* = .07. Any repetition effects for Unfamiliar faces did not approach significance, *F*s < 2.6, *p*s > .13.

Finally, it is noteworthy that most of the above ANOVAs also showed a reliable main effect of familiarity. Indeed, when unprimed familiar faces were contrasted directly against unprimed unfamiliar faces, the potentials were reliably more positive for the familiar faces, *F*(1,11) = 7.18, *p* < .05. Thus repetition of familiar faces appears to increase the size of a late centroparietal positivity associated with familiarity of a face.

In summary, this pattern for the late ERP repetition effect resembled the pattern of behavioural priming: reliable for familiar faces, but not for unfamiliar faces, and greater for familiar faces repeated with the same than different view.

### Latency analysis

To estimate the onset of the repetition effects, ANOVAs were repeated over successive 10 ms time bins from − 200 to + 600 ms poststimulus, and any reliable effects at *p* < .05 involving the repetition factor over at least three successive time bins were noted. The ANOVA over the four parietal-occipital channels where the early effect was maximal (see above) showed effects of global repetition in every 10 ms segment from + 80 ms to + 170 ms. The ANOVA on the three parietal channels where the late effect was maximal showed an effect of global repetition for familiar faces in every 10 ms segment from + 340 ms to + 450 ms.

### Effects of prime visibility

As with the RT data, further regression analyses against the *d*′ scores for prime visibility for each participant were performed on the early and late ERP effects. The results for the channels showing early (P7) and late (Pz) repetition effects are shown in Figs. 2B and C, respectively. For the early global repetition effect at P7, collapsed across familiar and unfamiliar faces, the intercept was reliably different from zero (95%CI = − 1.72 to − 0.42), as with the behavioural data in Fig. 2A. For the late global repetition effect for familiar faces, the intercept did not quite reach significance (95%CI = − 0.04 to 1.67), though there was no indication that the size of the effect increased with higher *d*′ values.

A 3D SPM analysis was also performed on contrast images corresponding to the above global repetition effects, in which *d*′ was included as a covariate of no interest. The test on the global repetition contrast collapsed across familiar and unfamiliar faces included pixels in bilateral temporoparietal clusters during the early time period (e.g., *x* = − 29, *y* = − 17, *t* = 120 ms; *Z* = 3.47; *x* = + 23, *y* = − 17, *t* = 114 ms; *Z* = 3.85) that survived correction for the pixels that showed a main effect of repetition in the earlier SPM analysis. The test on the global repetition contrast for familiar faces included pixels in a centroparietal cluster in the later time period (e.g., *x* = − 11, *y* = − 17, *t* = 380 ms; *Z* = 4.21) that survived correction for the pixels that showed a simple effect of repetition in the earlier SPM analysis. Thus neither early nor late repetition effects appear explicable by *d*′.

### Other potential causes

**Fig. 6:**
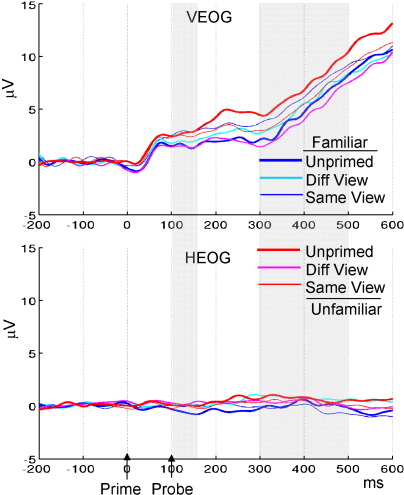
Vertical (top) and horizontal (bottom) EOG for each condition.

One potential confound of the above ERP effects is eye movement. There were no reliable condition effects on the HEOG in either the early or late time window. There were, however, main effects of repetition in the VEOG that was either marginal for the early time window, *F*(1.72,18.9 = 3.54), *p* = .06, or reliable for the late time window, *F*(1.43,15.7) = 4.43, *p* < .05 (Fig. 6). Small eye movements are often difficult to avoid when viewing a face (despite instructions to fixate). It is possible that such eye movements contributed to the ERP effects (though, of course, it is also possible that neural activity caused the changes picked up on the EOG electrodes). Eye movements are unlikely to be the sole cause of the early and late repetition effects, because the pattern of VEOG potentials across conditions differed from that of the repetition effects (e.g., on P7 or PZ), and the polarity of the ERP effects over posterior channels reversed across early and late time windows, whereas that of the VEOG effects did not. Nonetheless, we address this issue more fully in the within-trial analysis below.

Another potential contribution to the late repetition effect is neural activity related to motor preparation, given that the mean RT of approximately 600 ms was not much longer than the end of the late time window (500 ms). In other words, the late ERP effect could reflect neural processes downstream of those related to face recognition.

A third potential contribution we considered was the possibility that the ERP repetition effects, particularly the early one, were related to the degree of visual “overlap” between the prime and probe image. We therefore calculated the “visual dissimilarity” between the prime and probe images for each trial. Dissimilarity was defined as the root mean square difference across pixel grey levels in the two images, having first normalised each pixel grey level by the mean value per image (see, e.g., Vuilleumier et al., 2002). Identical images would have a value of 0. As expected, the mean dissimilarity was greatest for unprimed conditions, and least for Same-View conditions (see Table 1). Importantly, however, there was considerable variability across trials (prime-probe pairs) within each condition, which allowed some leverage in separating the effects of visual dissimilarity from the condition effects.

To test whether eye movements, motor preparation, and/or visual dissimilarity contributed to the ERP repetition effects, the SPM analysis was repeated, but this time, four covariates were added to the 1st-level model that was used to estimate the mean ERP images across trials for each condition and participant. These covariates were: mean VEOG amplitude from 100 to 150 ms, mean VEOG amplitude from 300 to 500 ms, Reaction Time, and Visual Dissimilarity. For each participant, the ERP images therefore reflected the mean potential having covaried out trial-to-trial variability in these four variables.

The resulting SPM analyses across participants showed that both the main effect of priming during the early time period and the simple effect of priming for familiar faces during the late time period remained reliable (see Table 2). This suggests that these variables cannot explain fully the ERP repetition effects observed.

## Discussion

Our data show two ERP effects associated with sandwich masked face repetition: an “early” repetition effect occurring for both familiar and unfamiliar faces, and a “later” effect occurring only for familiar faces. The amplitude pattern for the early effect resembled that found in our previous fMRI study, in that it was found for both familiar and unfamiliar faces, though there was a significant modulation by view (with greater deflections for same than different views, at least for familiar faces), which was only a numerical trend in our fMRI data. The amplitude pattern for the late effect, on the other hand, resembled the concurrent behavioural priming. Importantly, neither the ERP nor behavioural effects appeared to be driven by prime visibility, suggesting that they reflected subliminal processing. Nor did either ERP effect appear to be an artefact of pixel-wise visual overlap, overall reaction times, or eye movements.

### Subliminal face priming?

This is one of several experiments in which we have found evidence of RT priming for sandwich masked familiar faces that cannot be easily explained by measures of prime visibility (Henson and Kouider, 2007). In some of these experiments, we found greater priming when the same vs. a different view of a familiar face was repeated, in others we found priming of similar size for same and different views, but in all cases priming remained reliable even across different views. This suggests that the processes contributing to the behavioural facilitation operate at a certain level of abstraction. Our finding that this behavioural effect only occurs for familiar faces suggests that access to pre-existing face representations, or even to semantic information, may be necessary. However, this finding could also be a consequence of the specific task used, i.e., fame judgments. We used this task because it gives large priming effects under more conventional, “supraliminal” conditions (Ellis et al., 1990); however, it is possible that the reduced priming for unfamiliar faces in this task reflects a feeling of familiarity induced when the prime matches the probe (Jacoby and Whitehouse, 1989), which interferes with the fame judgment to the probe (counteracting any facilitation of, for example, perceptual processing).

One factor that might affect the present behavioural priming is the fact that stimuli were repeated across trials, i.e., each face appeared 6 times as a visible probe across the experiment. Repetition of stimuli as visible targets allows, for example, the development of direct, stimulus–response associations, which are known to play a role in many studies of masked priming (Damian, 2001). However, it is not clear how such associations could explain the priming found here, given that the prime and probe in our “unprimed” condition also required the same response. In addition, it is noteworthy that we have found subliminal face priming even for faces presented only once throughout an experiment (unpublished data).^4^

Nonetheless, one should be cautious before claiming that the present behavioural and ERP repetition effects are truly subliminal. In the final “visibility” test, though participants could not categorise the prime very accurately, they could (on average) do better than chance. Though this performance was only assessed after participants were instructed about the presence of the prime and explicitly oriented towards it, it remains possible that participants also had some awareness for the fame status of the prime on some trials of the main experiment. Though we have used the linear regression technique of Greenwald et al. (1995) to argue against this possibility, this technique is not perfect (e.g., incidental awareness of primes during the main experiment may not correlate with participants ability to “see” primes when explicitly instructed, and the relationship between priming and d’ may be nonlinear). A trial-by-trial measure of priming, ERP, and prime visibility might be informative in this regard.

### The “early” ERP masked face repetition effect

The early ERP effect was a more negative potential at bilateral temporoparietal channels for primed faces that onset around 80 ms, was maximal around 120 ms, and over by approximately 180 ms. This latency encompasses that of the typical P1 visually evoked response, though this component is less easily interpreted in the present context of a number of evoked responses to successive visual events (i.e., onsets of prime face, backward mask and probe face). It is interesting to note that this repetition effect onsets earlier than would an N170 response to the probe face, a component that has been long associated with face perception (Bentin et al., 1996; Botzel et al., 1995; also known as the VPP, Jeffreys, 1996) and modulations of which have been demonstrated for (unmasked) faces repeated within 800 ms (Harris and Nakayama, 2007; Jacques et al., 2007). It is also interesting that the early effect is not within the time window of the N250r, another repetition effect that has been consistently reported for unmasked faces repeated immediately across trials (with SOAs of a few seconds; Schweinberger et al., 2002). However, the effect is within the time window in which an N250r might be observed in response to the prime face, rather than probe face (i.e., 200–250 ms post-prime onset; see also Trenner et al., 2004). This is analogous to the observation here and by Jeffreys (1996) that a putative N170/VPP appears to coincide with the onset of the first rather than second of two faces presented in quick succession. It is possible then that the present early repetition effect reflects a modulation of processing of the prime face (e.g., a curtailment of processing in the unprimed condition) that occurs subsequent to the N170 response to that face, but at the same latency of processing that would be modulated by an unmasked face occurring several hundred milliseconds earlier, as in previous demonstrations of the N250r.

More generally, the fact that the present masked repetition effect is earlier (relative to the probe onset) than found with SOAs of a second or more (Harris and Nakayama, 2007; Schweinberger et al., 2002) might suggest that the effect occurs during an initial, predominantly feedforward passage of neural activity (reflecting, for example, interactions within regions owing to residual activity evoked by the prime). This could be distinct from long-lag priming effects, which have been hypothesised to reflect recurrent activity modulated by synaptic changes in top-down predictions from regions higher in the cortical processing pathway (Henson, 2003). Note also that the present repetition effects are more likely to reflect “subliminal” than “preconscious” face perception. “Subliminal” perception is believed to reflect bottom-up neural activity whereas “preconscious” perception is believed to reflect recurrent but local cortical loops (Dehaene et al., 2006; Kouider and Dehaene, 2007). Preconscious perception occurs for supra-threshold stimuli that remain unconscious because of inattention, whereas subliminal perception occurs for stimuli that remain invisible even under conditions of focused attention, which was likely for the majority of the subjects in this study (see Kouider et al., 2007, for fMRI evidence for a distinction between subliminal and preconscious perception of words).

A similar if not identical early ERP effect was reported for sandwich masked faces by Martens et al., (2006; Experiment 1), though they examined only same-view, famous faces. In the masked, short SOA condition of this experiment, the prime face was presented for 34 ms, and the backward masks were scrambled images. A subsequent experiment using a prime–probe matching task found performance levels that did not differ from chance, suggesting that prime visibility was minimal under these conditions (though visibility was not explicitly related to the ERP effects). Surprisingly, however, these authors did not find reliable behavioural priming (in a binary semantic judgment about the occupation of the famous people). This may be related to the fact that these authors did not find any later ERP repetition effects for their masked primes, such as the late effect discussed below, or such as the later repetition effects they did report for an unmasked version of their paradigm (which was associated with behavioural priming).

Eddy et al. (2006) also found repetition effects for masked greyscale images of objects during a target detection task, in which the target was defined semantically (a food item). This task did not allow a behavioural measure of priming, but given that the target could appear as the prime, it did allow an index of prime visibility (their mean *d*′ was 0.81). While prime visibility was not essential to their conclusions, their ERP data included an effect that onset around 100 ms and was maximal around 190 ms, somewhat later than the “early” effect found here (and with a slightly more posterior, occipital focus). Nonetheless, it is likely that these ERP effects reflect similar processes, differing perhaps in the nature of the stimuli (objects vs. faces).

The precise nature of the face processes modulated by repetition during the time window of the present early ERP effect deserves further investigation. The present data suggest that they are likely to be relatively low-level visual processes, in that the effect was found for both familiar and unfamiliar faces (when presented in the same view), and was sensitive to the precise view of a particular face (particularly for familiar faces). The critical processes might correspond to the structural encoding stage of the Bruce and Young (1986) model, though we have no direct evidence that the processes are specific to faces. Nonetheless, they do not appear to operate over iconic representations, in that the ERP effect was not explicable by a pixel-level measure of visual overlap between prime and probe image. It is less clear why the ERP effect showed greater sensitivity to view for familiar than unfamiliar faces (though it could reflect the greater variability in visual dissimilarity across different views of familiar faces, relative to different views of unfamiliar faces—Table 1). In future studies, we aim to investigate these issues further, using more controlled manipulations of view to explore the extent to which faces are processed according to the presence or absence of perceptual awareness.

Given the similarity of the early repetition effect with our fMRI data (Kouider et al., in press), in terms of the amplitude differences across conditions, it would seem likely that the generators of the early effect are in posterior occipital or fusiform cortex, or possibly posterior superior temporal sulcus. It is interesting that it is this relatively short-lived “early” ERP effect, rather than the relatively longer “late” ERP effect, that seems to match better the fMRI data (unlike the situation reported for attentional modulation of face processing by Furey et al., 2006).

### The “late” ERP masked face repetition effect

The late effect was a more positive potential over centroparietal channels for primed faces that onset around 340 ms and was maximal around 400 ms, close to the peak of a positive component. It was reliable only for familiar faces, as with the behavioural priming. Effects in a similar time window have been reported for masked objects (Eddy et al., 2006) and for masked words (Kiefer, 2002; Holcomb and Grainger, 2006; Woollams et al., in press). In the report of Eddy et al. (2006), the repetition effect was related to modulations of the N300 and N400 components. In the case of the N400, the repetition effect was attributed to semantic processing, given that N400 repetition effects appear to be sensitive to semantic variables (e.g., Chwilla et al., 1995). A semantic locus would be consistent with demonstrations of semantic processing of faces for which participants are minimally aware (Stone and Valentine, 2007). It would also be consistent with the present findings that (1) the ERP repetition effect occurred for familiar but not unfamiliar faces, and (2) a similar ERP effect occurred even when comparing unprimed familiar faces with unprimed unfamiliar faces.^5^ However, both these findings could also reflect target effects within the present fame-judgment task, rather than face familiarity per se (see earlier discussion of behavioural priming). Alternatively, the effect could arise at the level of long-term perceptual representations of known faces (e.g., FRUs in the Bruce and Young, 1986, model). These two possibilities could be distinguished by examining ERP correlates of masked priming from famous names to famous faces.

While the markedly different scalp distributions for the early and late masked repetition effects suggest different distributions of cortical activity, we can say little about the generators of the late ERP repetition effect on the basis of our previous fMRI study (no repetition effects restricted to familiar faces were found in Kouider et al., in press). One possible candidate is the anterior ventral temporal lobe, such as rhinal cortex, which has been shown by intracranial ERP studies to produce an N400-like response to familiar relative to unfamiliar faces that is further modulated by repetition (e.g., Trautner et al., 2004). We did not attempt source localisation of the present data because of the problem in obtaining an accurate forward model for EEG (given unknown conductances of different tissue types; and also the lack of MRIs and electrode digitisation for the present participants). An MEG version of this experiment may allow better localisation.

## Conclusion

In conjunction with our previous fMRI findings, the present ERP data suggest that facial identity can be processed unconsciously in the brain to some degree of abstraction (at least under conditions of temporal and spatial attention, as in the sandwich-masked paradigm). The ERP data have shed light on these processes by revealing at least two, dissociable effects that emerge over time. One onsets as early as 80 ms after the appearance of a primed face, and appears to mirror the fMRI repetition effects in occipital/fusiform regions, implicating rapid perceptual processing, though at a level above simple visual overlap. The other occurs later, appears to mirror the pattern of behavioural priming, and may reflect semantic processing. However, the precise nature and extent of this processing of stimuli for which participants are minimally aware remain an important question for future research.

## Figures and Tables

**Table 1:** Reaction times (RTs) for correct fame judgments for each condition, together with measure of prime–probe “Visual Dissimilarity” (Vis Dis)

Condition	Familiar same-view	Familiar different-view	Familiar unprimed	Unfamiliar same-view	Unfamiliar different-view	Unfamiliar unprimed
RT (ms)	594 (64)	602 (56)	615 (51)	640 (54)	648 (58)	647 (58)
Vis Dis/10^2^ (min = 0)	2.67 (0.25)	2.99 (0.66)	3.26 (0.80)	2.74 (0.29)	2.84 (0.51)	3.17 (0.85)

SD in brackets, either across participants for RT, or across trials (within participant) for Vis Dis.

**Table 2:** SPM “space-time” results for omnibus ANOVA effects, FWE-corrected *p* < .05

	*N*	*x* (*θ*)	*y* (*φ*)	*t*/ms	*Z*	Nearest channel
*ANOVA effect*						
Repetition	875	+ 20 (+ 75)	− 20 (− 50)	124	6.17	P6/P8
	− 29 (− 83)	−17 (+ 40)	120	5.64	P7
	+ 14 (+ 76)	− 26 (− 65)	100	5.61	P04
	− 17 (− 76)	− 23 (+ 55)	120	5.44	P5
	− 8 (− 87)	− 32 (+ 77)	102	5.51	O1
Familiarity	3715	− 17 (− 45)	− 2 (+ 30)	494	7.05	CP3
	− 11 (− 32)	− 2 (+ 41)	344	5.67	CP1
Repetition (Familiar faces)	43	− 8 (− 42)	− 8 (+ 63)	378	4.22*	P1
23	+ 5 (+ 43)	− 11 (− 75)	424	4.02*	P2

*After covarying VEOG, RT, Vis Dis*						
Repetition	121	+ 20 (+ 75)	− 20 (− 50)	126	5.71	P6
44	− 26 (− 79)	− 17 (+ 43)	120	5.50	P5
Repetition (Familiar faces)	34	− 5 (− 39)	− 8 (+ 70)	378	3.72*	P1

The origin of SPM coordinates is midpoint of a square image, with *x* ranging from − 48 (left) to + 48 (right) and *y* ranging from − 39 (posterior) to + 45 (anterior). The polar angles *θ* and *φ*, and the nearest channel, refer to the 3D space defined by Easycap (based on the international 10–20 system); *t* refers to peristimulus time; *Z* = *Z*-score, *N* = number of “voxels”; Vis Dis = Visual Dissimilarity, * = Small-Volume Corrected (SVC) for the main effect of familiarity. Note that only the global maximum is shown for main effect of familiarity, which was not the main focus of the present study (other clusters showing a familiarity effect are available on request).

## Footnotes

1 Separate estimates of *d*′ for each type of visibility trial – i.e., trials with non-matching prime and probe fame, matching trials (same view), matching trials (different view) and matching trials (unprimed) (see Materials and methods) – did not differ reliably, *F*s < 1.

2 Note that ANOVAs including all 63 channels as a single factor showed essentially the same results.

3 For completeness, there was a reliable simple main effect of familiarity for the Same condition, *F*(1,11) = 9.37, *p* < .05, and Unprimed condition, though only on the right, *F*(1,11) = 8.43, *p* < .05, but not the Different condition, *F*(1,11) > 2.42, *p* > .14.

4 It remains possible that one or both ERP repetition effects, unlike the behavioural effects, require that the prime faces have been perceived consciously in the recent past, e.g., if processing of a masked prime is influenced by episodic traces established by prior, unmasked exposures (e.g., when that face appeared as a probe on a previous trial). However, it is worth noting that the late ERP repetition effect at least resembles what we have found in a masked word repetition priming paradigm in which some primes were never presented unmasked (Woollams et al., in press).

5 It is noteworthy that the familiarity effect for unprimed faces is earlier than we found in a previous study (Henson et al., 2003), though likely reasons include the use of a different task in that study, in which fame was incidental, and the fact that those faces had distinctive features like the hair removed.
